# Morphology Effect of Photoconverted Silver Nanoparticles on the Performance of Surface-Enhanced Raman Spectroscopy Substrates

**DOI:** 10.1021/acsomega.2c05958

**Published:** 2023-03-27

**Authors:** Carlos Puente, Nayely Pineda Aguilar, Idalia Gómez, Israel López

**Affiliations:** † Facultad de Ciencias Químicas, Centro de Investigación en Biotecnología y Nanotecnología, Laboratorio de Nanociencias y Nanotecnología, Universidad Autónoma de Nuevo León (UANL), Autopista al Aeropuerto Internacional Mariano Escobedo Km. 10, Parque de Investigación e Innovación Tecnológica, Apodaca 66629, Nuevo León, Mexico; ‡ Centro de Investigación en Materiales Avanzados, S.C. (CIMAV), Unidad Monterrey, Alianza Norte 202, Apodaca 66628, Nuevo León, Mexico; § Facultad de Ciencias Químicas, Laboratorio de Materiales I, Av. Universidad, Cd. Universitaria, Universidad Autónoma de Nuevo León, UANL, 66455 San Nicolás de los Garza, Nuevo León, Mexico

## Abstract

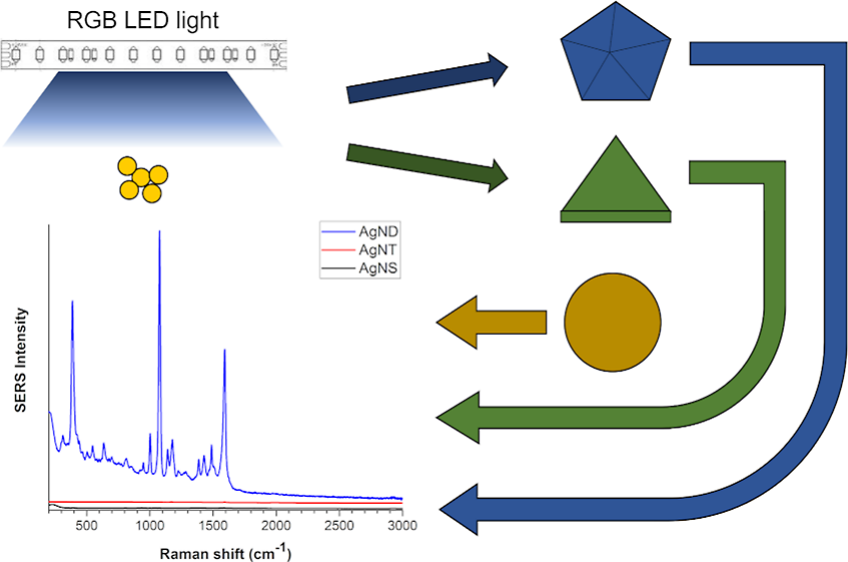

Nowadays, surface-enhanced Raman spectroscopy (SERS) substrates are of great interest for many researchers, aiming to fabricate substrates with high sensitivity and low fabrication costs. In this study, we photoconverted Ag nanoparticles by using a simple and affordable red–green–blue light-emitting diode photoreactor. The obtained dispersions were transformed into a paste of nanoparticles and used to fabricate SERS substrates by a simple drop-casting process under controlled humidity conditions. The performance of these substrates was tested using *p*-aminothiophenol as a Raman probe. The results indicate that the particle shape has an influence on the Raman intensities and substrate sensitivity, showing a significant enhancement as the number of faces and vertices in the particle increases.

## 1 Introduction

The constant search for new methods to analyze any kind of sample often leads to the improvement of existing technologies to achieve higher reproducibility and sensitivity. Raman spectroscopy has proven to be a powerful technique that allows the characterization of the sample taking advantage of its vibrational behavior. This behavior is specific for every molecule in a way that it can be considered its fingerprint.^1−5^ Therefore, the unique vibrational modes allow the differentiation of each sample component, including allotropes and polymorphic structures. However, the low intensity of the Raman signals often makes the unequivocal identification of the analyte difficult and thus a certain interpretation of the sample. An alternative technique that solves this problem is surface-enhanced Raman spectroscopy (SERS), which relies on the interactions among a nanostructured substrate, the analyte molecules, and the laser photons.^6,7^ It is known that the reproducibility and sensitivity achieved by this technique depend mostly on the substrates used during the analysis.^8,9^

A SERS substrate is composed of nanostructures deposited onto a suitable base such as any polished metal surface, glass, silicon, and even paper or a piece of cloth.^10,11^ The nanoparticles or structures used must be deposited uniformly along the substrate and close enough to produce hot spots. High electromagnetic field enhancements are generated at hot spots, leading to high Raman intensities,^12^ making the fabrication process a vital step that ensures a quality product. Many SERS substrate fabrication processes include the use of complicated or expensive techniques that provide batches with almost identical substrates such as electron beam lithography^13,14^ and laser ablation,^15,16^ among others. However, these procedures are often complicated and might be out of reach of many laboratories, technicians, and researchers. Due to this, cost-effective fabrication processes become more important, aiming to reach the same reproducibility at a lower production cost.

A second parameter to address is substrate sensitivity, which is directly related to the sample and nanoparticle characteristics. To increase the Raman intensities even at low concentrations, the functional material on the substrate must have a high interaction with the sample. There are methods to ensure and strengthen these interactions, such as changing the surface chemistry of the nanostructures to allow the detection of an analyte, increasing the specificity and sensitivity.^17^ However, for non-specific substrates, increasing the hot-spot formation and distribution is key for reaching a higher sensitivity. An increasing formation of hot spots directly allows a lower limit of detection, as hot spots increase the electric field around the nanoparticles, which is then translated as an enhancement in the Raman intensity. In this case, the nanostructure morphology plays an important role, as the presence of shapes with edges and tips induces a higher electromagnetic field enhancement at the hot-spot area.^18^

It is known that Ag nanoparticles can be converted or grown into different shapes and sizes, changing their plasmon modes and thus the properties of the obtained dispersion.^19,20^ Triangular nanoprisms (AgNT) are among the most used Ag shapes, along with nanocubes and spheres. These shapes can be obtained by a simple photoconversion, in which a nanoseed dispersion is irradiated by light to grow the particles into a different shape, using a variety of light sources, from light-emitting diodes (LEDs) to conventional fluorescent light.^21,22^

Nevertheless, the final shape may vary depending on the system parameters such as the capping agent, the presence of additional chemical species, the seed morphology, and the energy applied.^23,24^

Herein, we report the obtainment of nanostructures by photoconversion using an affordable red–green–blue (RGB) LED photoreactor and the fabrication of SERS substrates via paste drop-casting. The performance of each kind of substrate was evaluated using *p*-aminothiophenol (*p*-ATP) as a Raman probe in a concentration range of 10^–2^–10^–8^ M.

## 2 Materials and Methods

### 2.1 Materials

Silver nitrate (AgNO_3_), sodium citrate (Na_3_Cit), sodium borohydride (NaBH_4_), and *p*-ATP were of reagent grade and purchased from Sigma-Aldrich (Toluca, Estado de México, Mexico). Ethanol was used to prepare all *p*-ATP solutions, and deionized water was used for all other solutions.

### 2.2 Nanostructure Dispersion Obtainment

For the synthesis of nanosphere (AgNS) dispersions, 20 mL of 1 mM AgNO_3_ was placed in a beaker protected from any light source and heated until boiling conditions. Then, 400 μL of 1% w/v Na_3_Cit was added and left to react for 15 min at boiling temperature. The resulting orange–gray dispersion was centrifuged at 4500 rpm, washed with water, and stored in an amber vial.

To photoconvert nanoparticles to different morphologies, first, the Ag seed dispersion is obtained by mixing 58 mL of water, 10 mL of 1 mM AgNO_3_, and 30 mL of 5 mM Na_3_Cit and then adding 2 mL of 8 mM NaBH_4_ in a dropwise process to ensure the formation of small particles. The dispersion quickly changes from colorless to yellow when the NaBH_4_ drops react with the system. This seed dispersion is then stored and used with no further changes; its UV–vis spectrum can be found in the Supporting Information (Figure S1).

For the photoconversion process, test tubes with Ag seeds were set into a beaker with water, to maintain the temperature of the system, and set inside an LED photoreactor. This photoreactor contains a 12 V 5050 RGB LED strip that allows more control of the incident light wavelength range than an incandescent or fluorescent light. The particles are then irradiated for 24–48 h depending on the light wavelength. For Ag triangular nanoplates (AgNT), green light was used for 48 h, resulting in a blue dispersion, whereas for the obtainment of Ag nanodecahedra (AgND), blue light was irradiated for 24 h, leading to a pink–orange system. The photoconverted dispersions were purified by centrifuging at 8000 rpm, washing with water, and storing under dark conditions. The wavelength ranges reported by the LED manufacturer are 620–630 nm for red, 520–530 nm for green, and 465–475 nm for blue lights.

The Ag dispersions were characterized by UV–vis spectroscopy, and the nanoparticle morphology was characterized by transmission electron microscopy (TEM).

### 2.3 SERS Substrate Fabrication and Measurements

First, each dispersion is centrifuged to form a nanoparticle pellet or paste, which has a low water content. For the substrate fabrication, 3 μL of the paste is drop-casted onto mechanically polished Al substrates set into a high-humidity chamber and left to dry. In this case, a closed Petri dish with water inside was used to achieve high-humidity conditions. This allows a slow solvent evaporation and nanostructure assembly, reducing the coffee-ring effect and leading to uniform substrates. For each morphology, the SERS substrates were characterized by Raman spectroscopy with no sample (Supporting Information, Figure S2).

For the SERS measurements, 3 μL of a *p*-ATP ethanolic solution was drop-casted onto the nanostructure-containing substrates and left to dry before their analysis. To study the minimal detectable concentration for each kind of substrate, *p*-ATP concentrations were studied in a concentration range of 10^–8^–10^–2^ M. Each measurement was recorded at a single point; for each concentration or morphology, a new substrate was used. However, substrate and batch reproducibility was evaluated for AgND at 10^–8^, 10^–7^, and 10^–6^ M, which can be observed in the Supporting Information (Figures S3–S5).

### 2.4 Instrumentation

The optical properties of the obtained dispersions were characterized by a Mettler Toledo UV5 UV–vis spectrometer, and the particle morphology was observed using a transmission electron microscope, JEM-2200FS from JEOL. The SERS substrates were characterized by scanning electron microscopy (SEM) using a FEI Nova 200 NanoSEM microscope for the AgNS and AgNT substrates and a JEOL JSM-7401F microscope for the AgND substrates. The Raman spectra were recorded using a Thermo Scientific DXR Raman microscope with a 50X objective lens and a 780 nm laser at 2.2 mW and 10 s as integration time for AgNS and AgNT substrates and 1 s for the AgND-based substrates.

## 3 Results and Discussion

**Figure 1:**
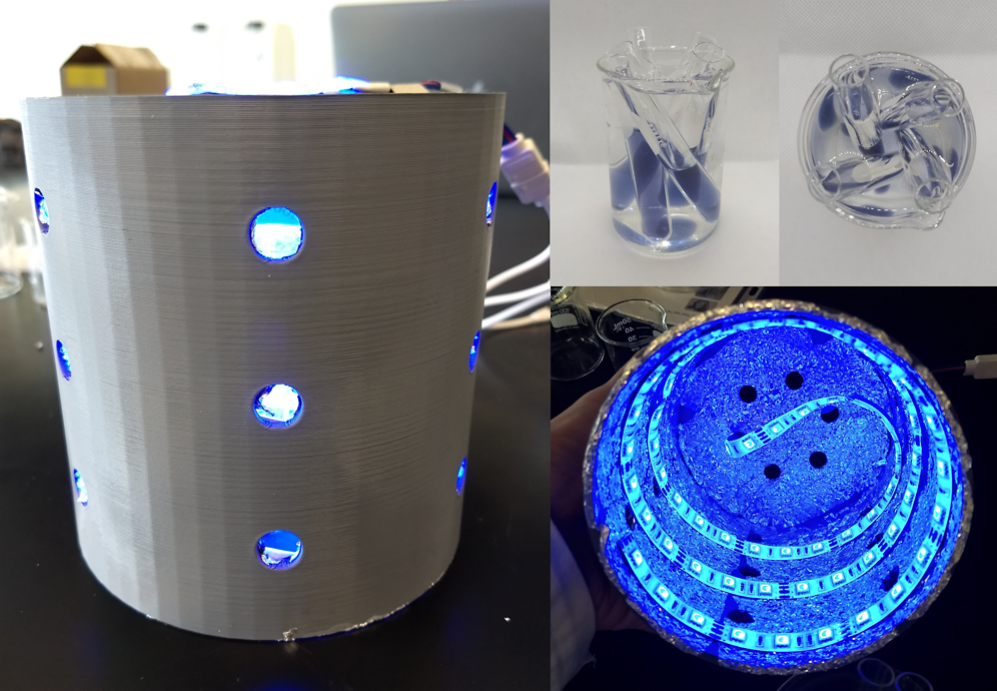
3D printed photoreactor with an LED strip used for the photoconversion of Ag nanoseeds into AgND and AgNT nanostructures.

To perform a photoconversion procedure, test tubes with Ag nanoseeds were exposed to light in the reactor shown in Figure 1. This reactor was 3D-printed to match specific measurements, and the interior was covered with tin foil to ensure a reflective surface. Also, the LED strip was oriented in a way that light interacts with the dispersions from side to side and from the top of the test tubes.

**Figure 2:**
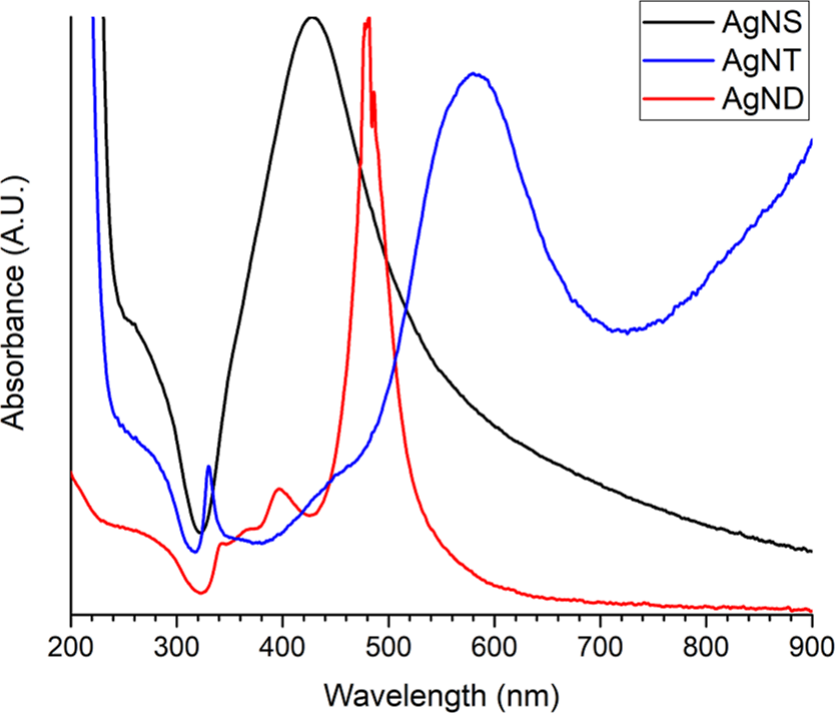
UV–vis spectra of the synthesized silver nanoparticle dispersions.

The optical properties of the dispersions obtained by photoconversion and direct chemical reduction were characterized by UV–vis spectroscopy as shown in Figure 2. It can be observed that the AgNS dispersion shows a single plasmon mode close to 425 nm. This plasmon mode corresponds to spherical particles obtained by a citrate method, leading to particles larger than the 7 nm nanoseeds.^25^ On the other hand, the optical behavior of the photoconverted dispersions is different from that of the original nanoseed system, indicating a shape change. The nanoparticles obtained by blue light irradiation show the typical plasmon modes of nanodecahedra, matching reports of Ag nanodecahedra obtained by blue LED irradiation.^26^ Lastly, the dispersion obtained by using green LED light for 48 h shows the characteristic behavior of Ag triangular nanoplates, a completely different shape synthesized by changing the irradiation wavelength. These spectra also give information about the most suitable substrate–Raman laser combination, as it is suggested that the wavelength of the laser used should be slightly blue-shifted from the strongest plasmon mode produced by the SERS substrate.^27,28^ However, for a uniform nanoparticle layer, the formation of hot spots along the active area and their unpredicted position on the laser spot allow the use of an excitation wavelength far from the localized surface plasmon resonance.^28^

**Figure 3:**
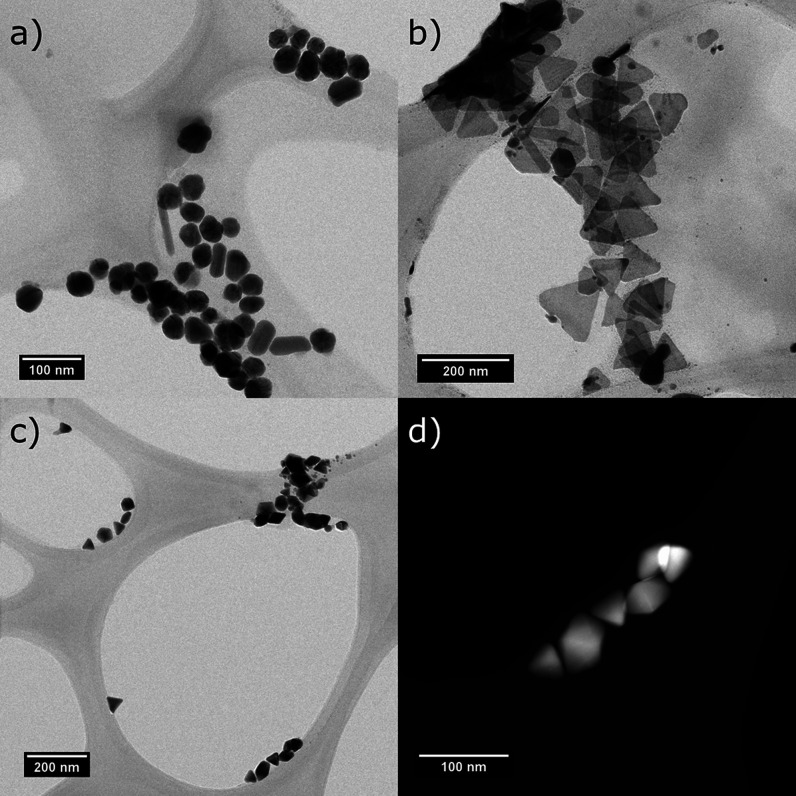
TEM micrographs of (a) AgNS, (b) AgNT, and (c,d) AgND dispersions.

On the other hand, the morphology of the nanostructures was characterized by TEM as can be observed in the micrographs shown in Figure 3. The AgNS micrograph shows the obtainment of spherical particles with a size of 38.7 ± 1.9 nm (average from 35 measured particles) and a small percentage of rod-shaped particles. This indicates a small contribution of this additional shape to the overall optical properties of the dispersions. These particles were not removed due to their size, which is similar to the average diameter of the nanospheres, leading to a similar behavior during the centrifugation purification.^29^Figure 3b shows the AgNT micrograph, in which triangular-shaped nanoplates with an average size of 92.6 ± 7.7 nm (average from 39 measured particles) can be observed and smaller nanoplates and nanospheres. It must be remarked that the triangular particles show rounded corners, which is a morphological parameter that impacts the hot-spot formation and distribution differently than particles with sharp edges. Lastly, in the case of the AgND dispersion, the micrograph indicates the formation of decahedral particles with an average size of 55.9 ± 3.0 nm (average from 16 measured particles), deposited in different orientations. Nevertheless, triangular structures can also be observed, which are usually related to decahedra due to their formation mechanism, as the seeds can grow into pyramids that assemble to form decahedra.^30^

**Figure 4:**
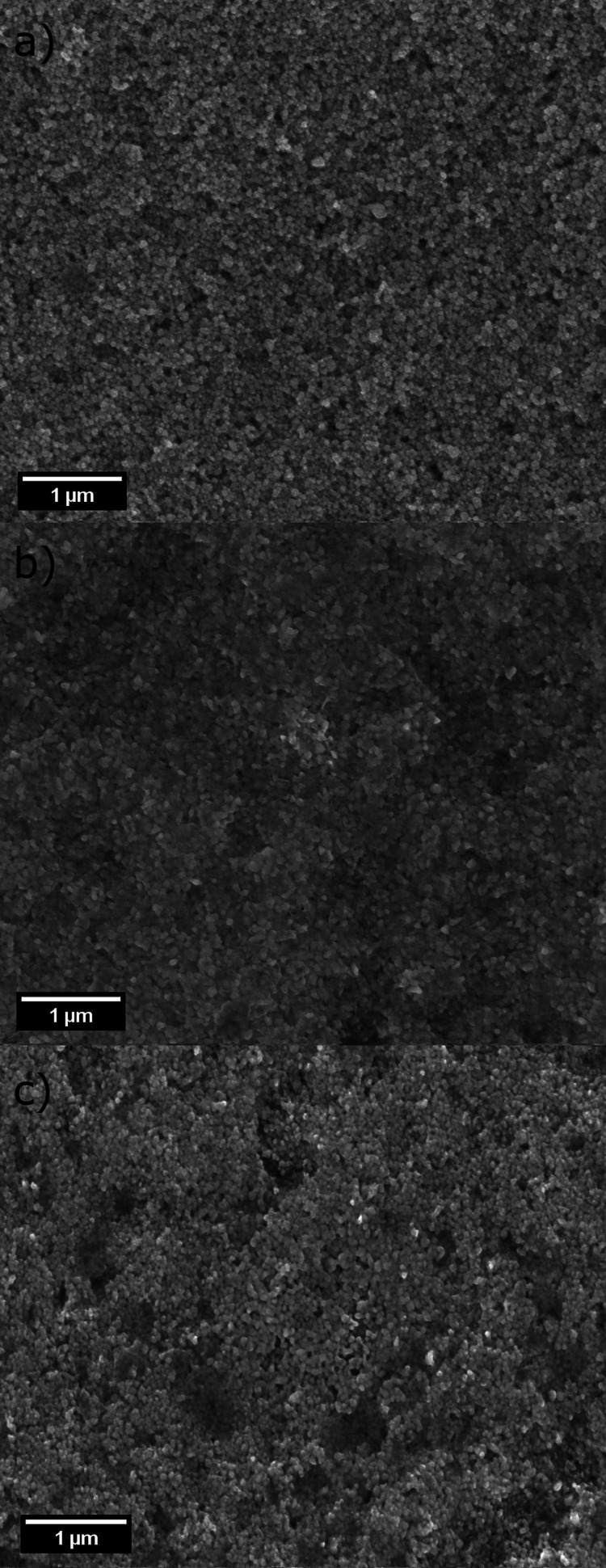
SEM micrographs of (a) AgNS, (b) AgNT, and (c) AgND substrates.

**Figure 5:**
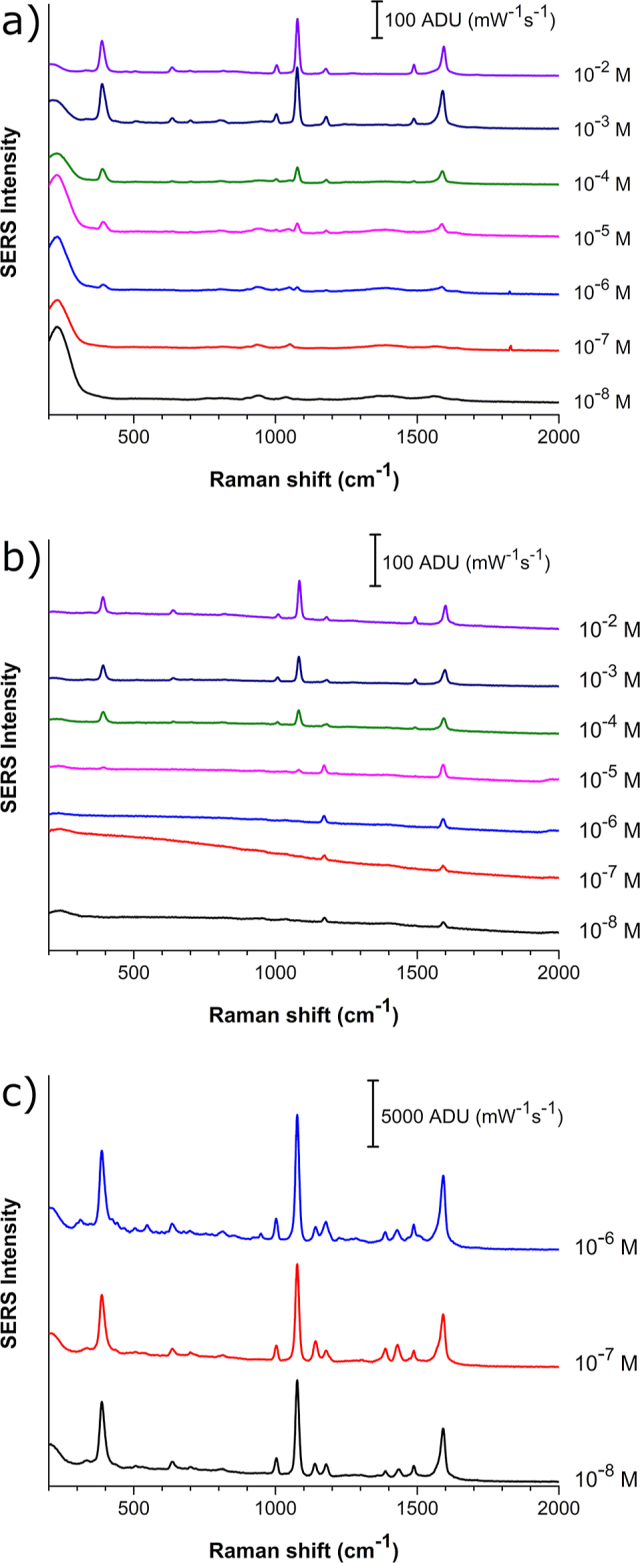
Raman spectra of *p*-ATP at different concentrations, recorded using (a) AgNS, (b) AgNT, and (c) AgND substrates.

The nanostructure dispersions were centrifuged to obtain a nanoparticle pellet which was used as a concentrated nanoparticle paste. This paste was then drop-casted onto polished Al substrates and left to dry under high-humidity conditions to reduce the coffee-ring effect and thus obtain uniform SERS substrates. The SERS substrates were characterized by SEM, and the obtained micrographs are shown in Figure 4. It can be observed that in all cases, a coating of nanostructures is formed by particles that are closely packed, forming a uniform distribution when compared with the laser area (1 μm). A SEM micrograph of a AgND substrate with higher magnification is available in the Supporting Information (Figure S6). These characteristics allow the balanced formation of hot spots throughout the substrates, which are parameters that impact reproducibility and Raman intensities. To test the performance of the substrates, *p*-ATP was analyzed as a Raman probe in a 10^–2^–10^–8^ M concentration range, as can be observed in the Raman spectra shown in Figure 5.

Figure 5a shows the Raman spectra collected using AgNS-based substrates, and it can be observed that the characteristic *p*-ATP Raman signals are detected at concentrations higher than 10^–5^ M. On the other hand, the substrates fabricated using the AgNT dispersion (Figure 5b) showed a lower SERS intensity compared to the AgNS-based substrates. Similarly, the sensitivity of the nanoprisms allowed the detection of *p*-ATP at concentrations as low as 10^–5^ M. Lastly, the AgND-based substrates (Figure 5c) showed a strong SERS effect, causing a signal saturation even at a concentration of 10^–8^ M, which was avoided by decreasing the integration time from 10 to 1 s. The results indicate that the morphology of the particles and their assembly on a substrate allow the formation of more hot spots compared to the other substrates, thus reaching higher sensitivity even at low concentrations. It was also observed that at higher concentrations, the intensities of the spectra obtained using AgNT and AgND substrates showed low increases compared to the 10^–8^ M analysis. This indicates saturation of the available sites in the SERS substrates. The latter occurs when a layer of probe molecules interacts with the nanoparticles at the substrate surface, acting as a barrier for the rest of the molecules. This barrier results in the quenching of the SERS interaction between the particles and the upper molecules. Even though this behavior is also shown in the AgNS substrates, the saturation is reached at higher concentrations.

The results obtained by SERS analysis illustrate the AgND substrate capability to detect a lower concentration of *p*-ATP when compared with that of the substrates based on the other morphologies. It can be observed that as the number of faces and vertices in the morphology increases, the SERS effect gets stronger and leads to higher sensitivity. The latter occurs due to the formation of more hot spots, whose effect depends on the interaction of the particles. In this way, the hot spot effect will be higher when they are formed by the interaction of two vertices. This behavior is easier to produce by using morphologies such as decahedra and other polygons.

## 4 Conclusions

In this study, we analyzed the effect of the nanoparticle shape on the performance of SERS substrates obtained by a cost-effective procedure. It was demonstrated that as the number of vertices and edges in the morphology increases, the SERS effect becomes stronger, which is caused by the presence of more hot spots throughout the substrate. This allowed us to fabricate substrates with high sensitivity, allowing a minimum detectable concentration of *p*-ATP of 10^–8^ M.

## Supporting Information Available

UV–vis spectrum of the Ag seed dispersion; Raman spectra of AgND, AgNT, and AgNS substrates with no sample; SERS spectra of 10^–6^–10^–8^ M *p*-ATP obtained at three different spots of three AgND substrates; and SEM micrograph of a AgND substrate (PDF)
