# Supplementary material for: Morphology Effect of Photoconverted Silver Nanoparticles on the Performance of Surface-Enhanced Raman Spectroscopy Substrates
Source: ACS Omega. 2023 Mar 27;8(14):12630–5. doi: 10.1021/acsomega.2c05958 (PMC10099118; doi:10.1021/acsomega.2c05958)
Supplement: Supplementary file 1 — ao2c05958_si_001.pdf [file ao2c05958_si_001.pdf]

Morphology effect of photoconverted silver nanoparticles on the performance of surface-enhanced Raman spectroscopy (SERS) substrates

Carlos Puente[a], Nayely Pineda Aguilar[b], Idalia Gómez[c], Israel López[a]*

[a]Universidad Autónoma de Nuevo León (UANL), Facultad de Ciencias Químicas, Centro de Investigación en Biotecnología y Nanotecnología, Laboratorio de Nanociencias y Nanotecnología, Autopista al Aeropuerto Internacional Mariano Escobedo Km. 10, Parque de Investigación e Innovación Tecnológica, 66629 Apodaca, Nuevo León, Mexico.

[b]Centro de Investigación en Materiales Avanzados, S.C. (CIMAV), Unidad Monterrey, Alianza Norte 202, 66628 Apodaca, Nuevo León, Mexico.

[c]Universidad Autónoma de Nuevo León, UANL, Facultad de Ciencias Químicas, Laboratorio de Materiales I, Av. Universidad, Cd. Universitaria, 66455 San Nicolás de los Garza, Nuevo León, Mexico.

*Email: israel.lopezhr@uanl.edu.mx

Contents

- Figure S1. UV-Vis spectrum of the Ag seed dispersion.
- Figure S2. SERS spectra of 10^{-3} M *p*-ATP obtained using the AgNS substrate, and Raman spectra of AgND, AgNT, and AgNS substrates with no sample.
- Figure S3. SERS spectra of 10^{-6} M *p*-ATP obtained at three different spots of three AgND substrates.
- Figure S4. SERS spectra of 10^{-7} M *p*-ATP obtained at three different spots of three AgND substrates.
- Figure S5. SERS spectra of 10^{-8} M *p*-ATP obtained at three different spots of three AgND substrates.
- Figure S6. SEM micrograph of a AgND substrate.

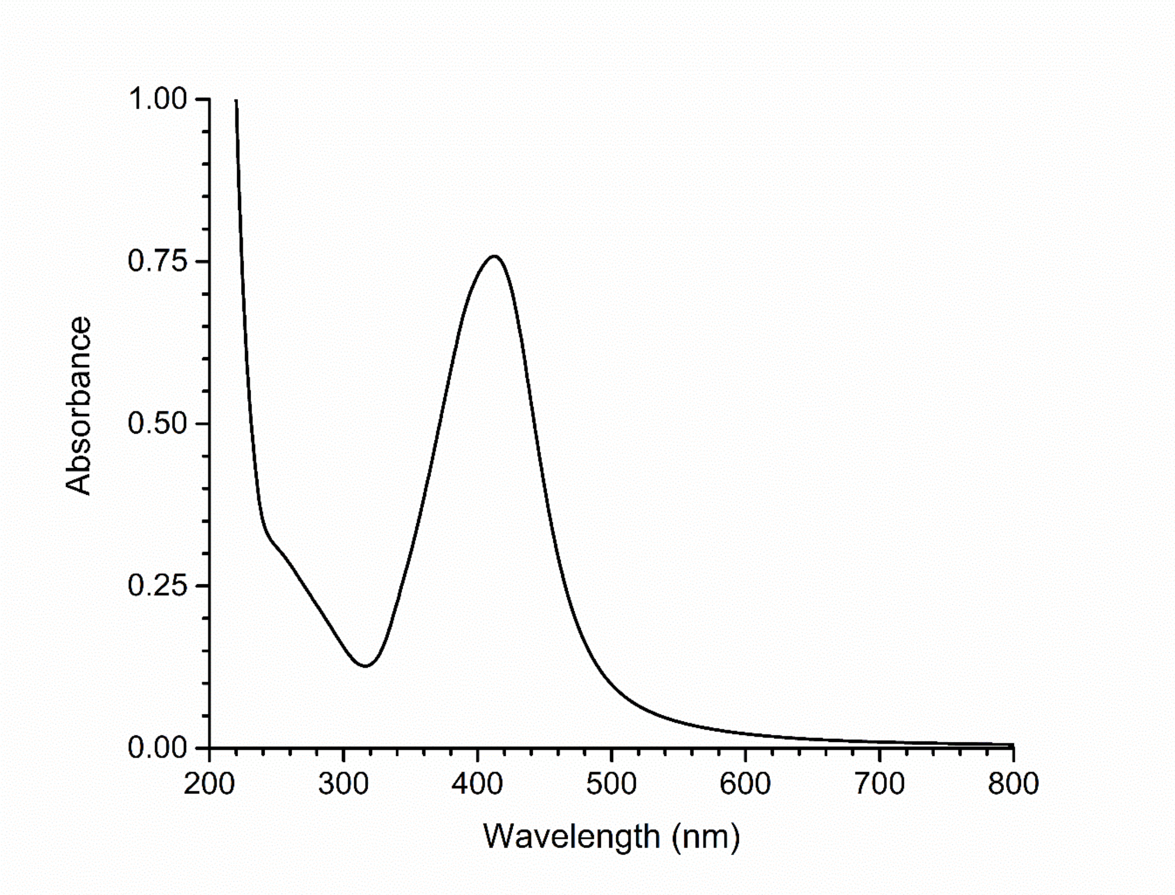

Figure S1. UV-Vis spectrum of the Ag seed dispersion.

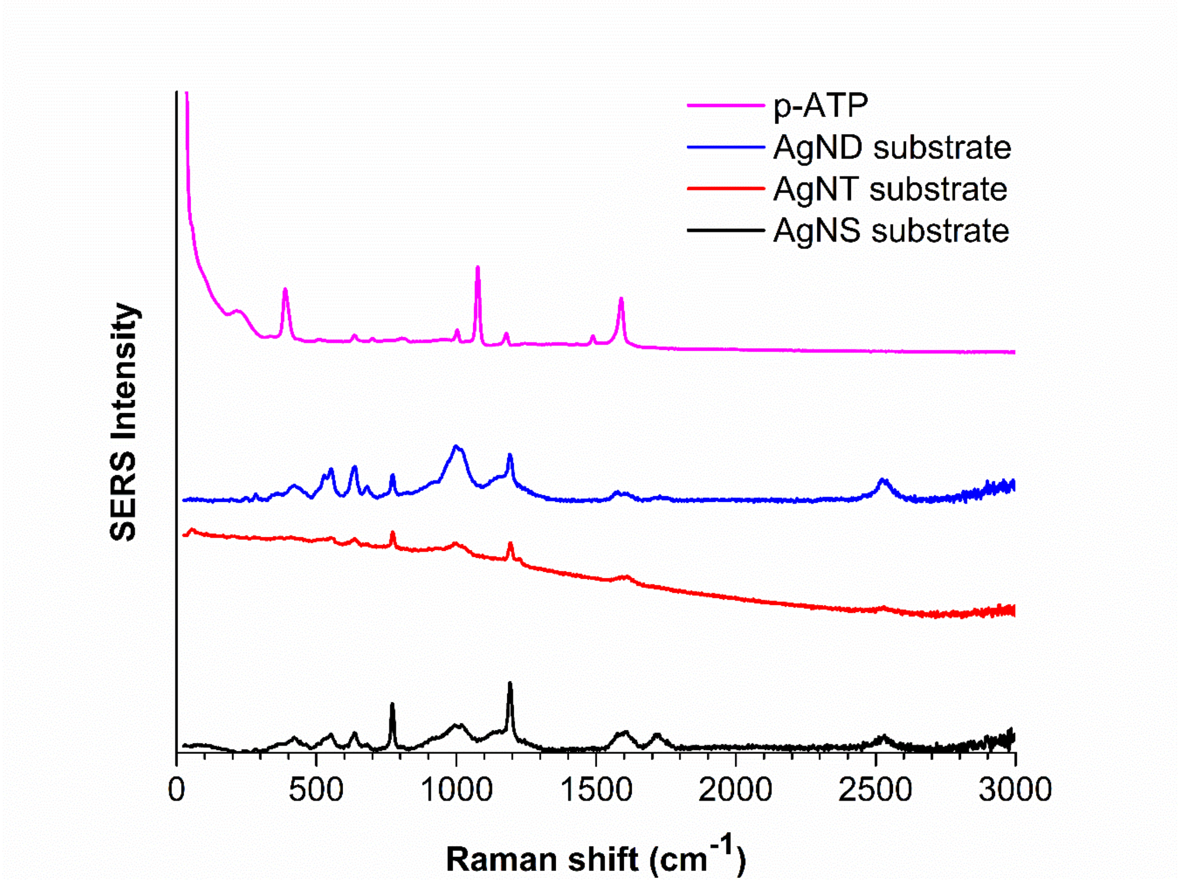

Figure S2. SERS spectra of 10^{-3} M *p*-ATP obtained using the AgNS substrate, and Raman spectra of AgND, AgNT, and AgNS substrates with no sample.

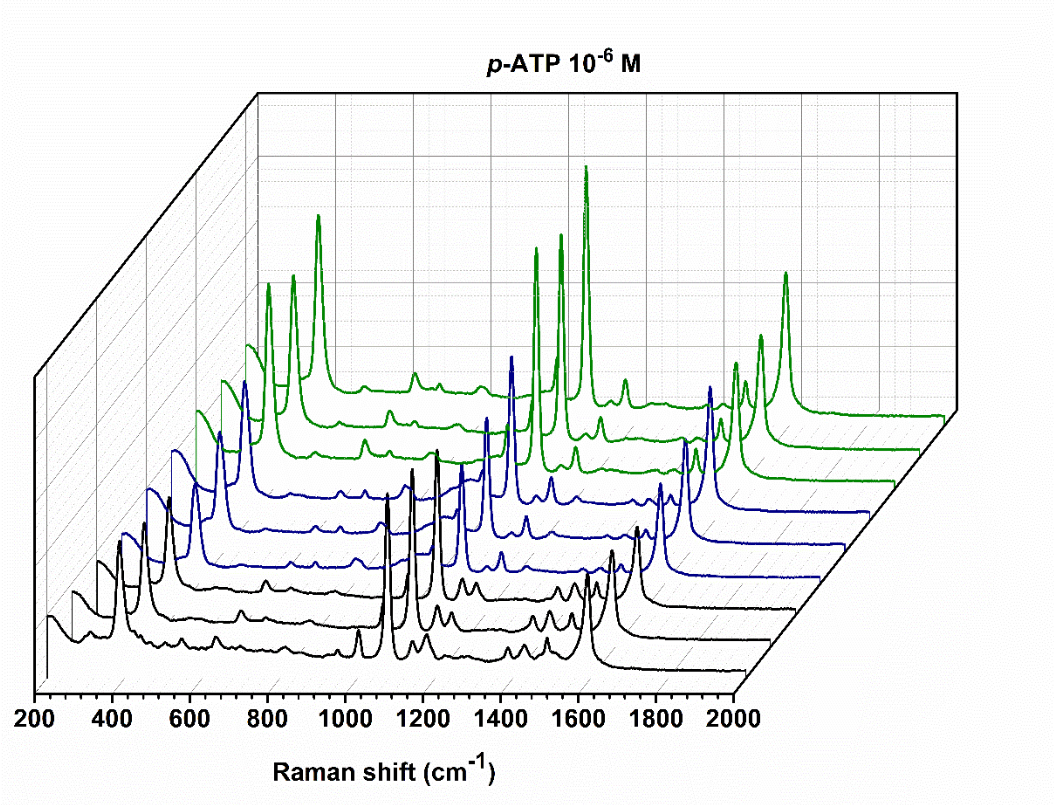

Figure S3. SERS spectra of 10^{-6} M *p*-ATP obtained at three different spots of three AgND substrates.

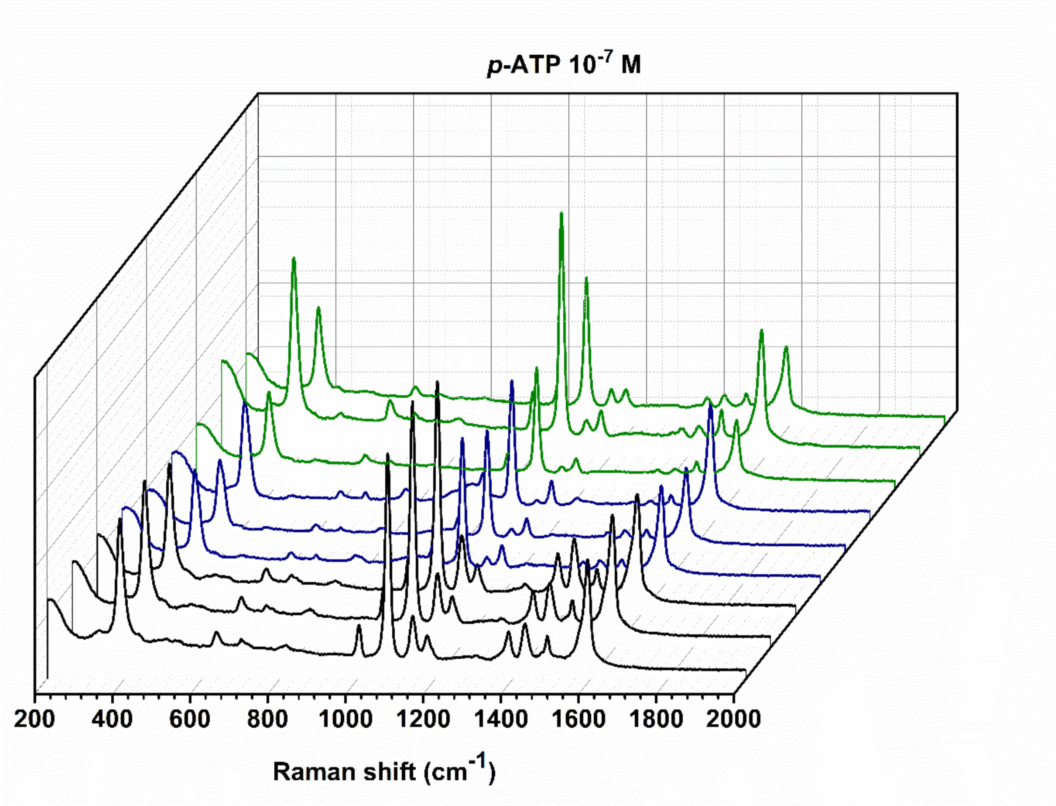

Figure S4. SERS spectra of 10^{-7} M *p*-ATP obtained at three different spots of three AgND substrates.

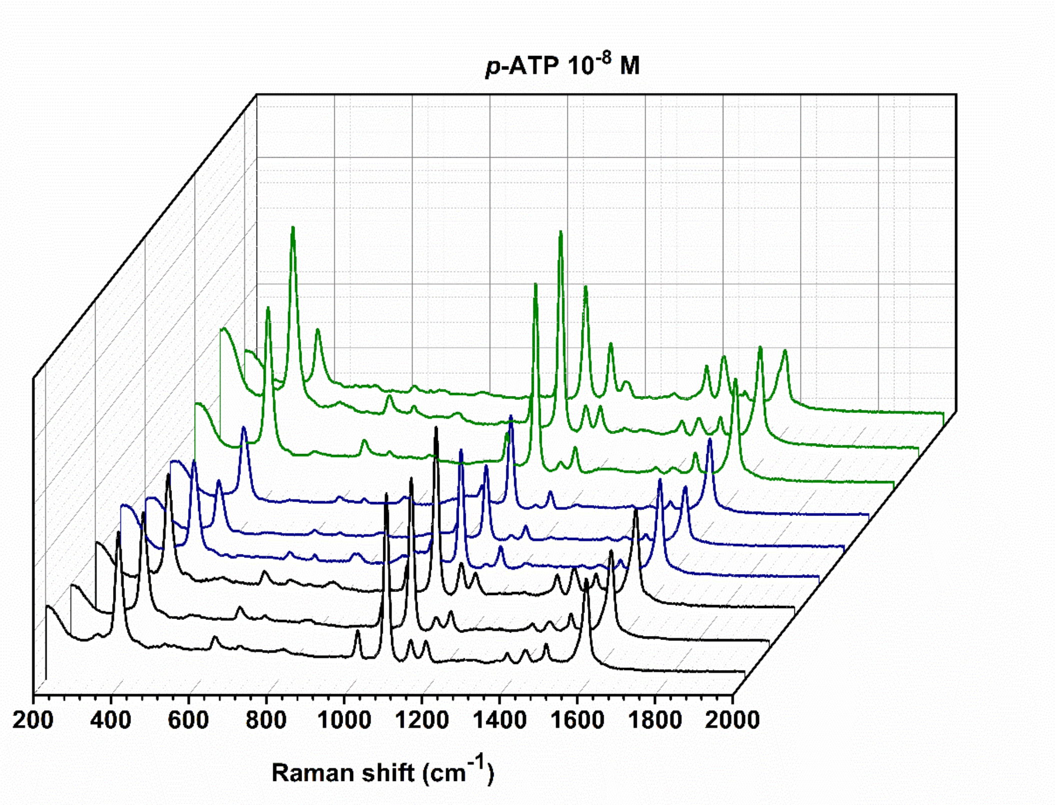

Figure S5. SERS spectra of 10^{-8} M *p*-ATP obtained at three different spots of three AgND substrates.

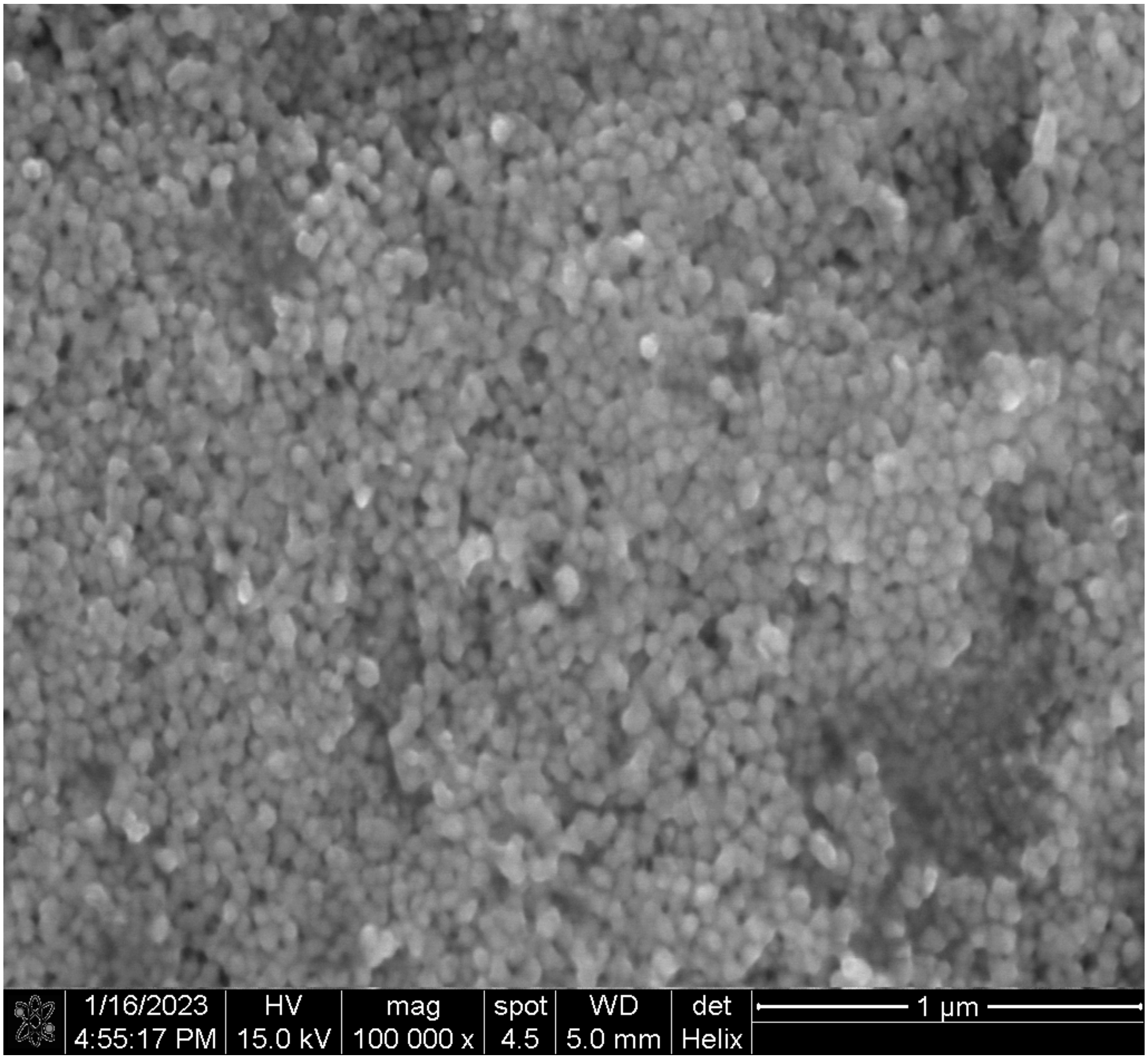

Figure S6. SEM micrograph of a AgND substrate.
